# The bidirectional relationship between chronic joint pain and frailty: data from the Investigating Musculoskeletal Health and Wellbeing cohort

**DOI:** 10.1186/s12877-023-03949-4

**Published:** 2023-05-05

**Authors:** Wendy J. Chaplin, Daniel F. McWilliams, Bonnie S. Millar, John R. F. Gladman, David A. Walsh

**Affiliations:** 1 Academic Rheumatology, Injury, Recovery and Inflammation Sciences, School of Medicine, University of Nottingham, Nottingham, UK; 2 Pain Centre Versus Arthritis, University of Nottingham, Nottingham, UK; 3 NIHR Biomedical Research Centre, University of Nottingham, Nottingham, UK; 4 Academic Rheumatology, Clinical Sciences Building, Nottingham City Hospital, Hucknall Road, Nottingham, NG5 1PB UK; 5 Centre for Rehabilitation & Ageing Research, Injury, Recovery and Inflammation Sciences, University of Nottingham, Nottingham, UK; 6 Sherwood Forest Hospitals NHS Foundation Trust, Rheumatology, Mansfield, UK

**Keywords:** Chronic pain, Frailty, Older people

## Abstract

**Background:**

Pain and frailty are associated, but this relationship is insufficiently understood. We aimed to test whether there is a unidirectional or bidirectional relationship between joint pain and frailty.

**Methods:**

Data were from Investigating Musculoskeletal Health and Wellbeing, a UK-based cohort. Average joint pain severity over the previous month was assessed using an 11-point numerical rating scale (NRS). Frailty was classified as present/absent using the FRAIL questionnaire. Multivariable regression assessed the association between joint pain and frailty, adjusted for age, sex, and BMI class. Two-wave cross-lagged path modelling permitted simultaneous exploration of plausible causal pathways between pain intensity and frailty at baseline and 1-year. Transitions were assessed using *t*-tests.

**Results:**

One thousand one hundred seventy-nine participants were studied, 53% female, with a median age of 73 (range 60 to 95) years. FRAIL classified 176 (15%) participants as frail at baseline. Mean (SD) baseline pain score was 5.2 (2.5). Pain NRS ≥ 4 was observed in 172 (99%) of frail participants.

Pain severity was associated with frailty at baseline (aOR 1.72 (95%CI 1.56 to 1.92)). In cross-lagged path analysis, higher baseline pain predicted 1-year frailty [β = 0.25, (95%CI 0.14 to 0.36), *p* < 0.001] and baseline frailty predicted higher 1-year pain [β = 0.06, (95%CI 0.003 to 0.11), *p* = 0.040]. Participants transitioning to frailty over one year had higher mean pain scores (6.4 (95%CI 5.8 to 7.1)) at baseline than those who remained non-frail (4.7 (95%CI 4.5 to 4.8)), *p* < 0.001.

**Conclusions:**

The bidirectional relationship between pain and frailty could lead to a vicious cycle in which each accelerates the other’s progression. This justifies attempts to prevent frailty by addressing pain and to include pain measures as an outcome in frailty studies.

**Supplementary Information:**

The online version contains supplementary material available at 10.1186/s12877-023-03949-4.

## Introduction

Frailty is a vulnerability state seen in older people due to multi-organ age-associated decline and is characterised by homeostatic failure in response to challenge [1]. Fifteen percent of people in the UK over 65 have frailty [2]. As frailty develops, people transition between robust, pre-frail and frail states. These transitions are not always permanent or unidirectional; people may transition from frailty or pre-frailty to more robust states. For example, The Irish Longitudinal Study on Ageing (TILDA) observed a frequent fluctuation between frailty states over an eight-year period [3], suggesting a dynamic aspect of frailty.

Pain is “an unpleasant sensory and emotional experience associated with, or resembling that associated with, actual or potential tissue damage” [4], and deemed chronic when pain lasts for more than three months [5]. Chronic pain is common, affecting between a third to half of the UK population’ [6]. Musculoskeletal conditions are particularly common causes of chronic pain, affecting over a third of the UK population. For example, it is estimated 9.5 million people in the UK have back pain [7]. As with frailty, chronic pain is also not necessarily a permanent state. For example, pain may reduce following exercise interventions for chronic low back pain [8].

Chronic pain is associated with frailty. A systematic review of prospective longitudinal studies showed people with chronic pain were twice as likely to become frail during the following year compared to people without pain [9], suggesting that chronic pain may play a causal role in the development of frailty. However, the relationship between chronic pain and frailty is complex. Chronic pain might make the transitions from non-frail to frail states more likely or make the transitions from frail to non-frail states less likely. Pain has been described as a marker of vulnerability and proposed as an additional criterion in frailty phenotyping [10]. Making this association yet more complex, the relationship between pain and frailty might be bidirectional: frailty states might cause chronic pain [11]. Unravelling these associations would be helpful, for example, to justify whether to address pain aiming to prevent the development of frailty, to treat pain aiming to reverse frailty, or to manage frailty aiming to prevent or reduce pain.

We aimed to test whether there is a unidirectional or bidirectional relationship between joint pain and frailty. To do so, we used a cross-lagged path analysis of a cohort of older adults followed over one-year. Cross lagged path analysis takes account of the relative strengths of bidirectional relationships to allow observers to infer the direction of causality or the degree of bidirectionality.

## Methods

### Participants and data sources

This study is an analysis of baseline and 1-year data from a subgroup of participants in the Investigating Musculoskeletal Health and Wellbeing (IMH&W) study; an ongoing longitudinal cohort based in the East Midlands, UK [12].

IMH&W recruited, through multiple pathways, adults with or at risk of musculoskeletal problems or frailty. General Practitioners (GP) recruited patients with an electronic Frailty Index (eFI) score of ≥ 0.12 (the threshold for mild frailty) [13]. The eFI is used by UK healthcare providers composed of 36 deficits, consisting of comorbidities, symptoms, activity/ mobility restrictions, social vulnerability, and care requirements [13]. IMH&W also recruited from participants in previous University of Nottingham research studies. Ethical approval for IMH&W was by the Central London Research Ethics Committee (ref.18/LO/0870).

At the time of screening for the current study, 5,500 participants had completed baseline IMH&W questionnaires, providing data on demographics, medical conditions, medications, information on joint aches and pains, and information on activities and general health, and incorporating the FRAIL questionnaire [14]. A CONSORT diagram is shown in Additional Fig. 1 to indicate the number of participants at each stage.

The inclusion criteria for the current study required participants to be aged ≥ 60 years at baseline and have completed all items of the FRAIL questionnaire at both baseline and 1-year. Data were extracted in July 2020.

### Measures

The FRAIL questionnaire comprises five self-report items: **F**atigue (tired all or most of the time in the last 4 weeks = 1, from a 5-point Likert scale); **R**esistance (difficulty climbing 10 steps without aids or rest) and **A**mbulation (difficulty walking several hundred yards without aids) each scored as 0: no or 1: yes; **I**llnesses (< 5 = 0, ≥ 5 = 1, from 11 specified morbidities) and **L**oss of weight (< 5% = 0, > 5% in a year = 1) [14]. Illness counts were determined based on self-report using a checklist of diagnoses with the question; ‘has a doctor told you that you have any of these conditions or problems’: angina, arthritis, asthma, cancer (not minor skin), chronic lung disease, congestive heart failure, diabetes, heart attack, hypertension, kidney disease, and stroke. Weight loss at baseline was calculated in response to two questions, firstly, ‘one year ago how much did you weigh without shoes but with your clothes on?’ and secondly, ‘how much do you weigh with your clothes on but without shoes?’. Percentage weight change was calculated as [(weight 1-year ago – current weight)/ weight 1-year ago] × 100. Each item was scored as 0: not present or 1: present. The total score classifies participants into robust (0), prefrail (1 or 2) and frail (3 to 5). For the current study, robust and prefrail groups were combined to give a non-frail category. FRAIL was dichotomised to ensure that clinical frailty was present rather than mobility-related issues which may be found in a pre-frail group.

Joint pain intensity was measured using a numerical rating scale (NRS). In this paper any reference to pain NRS refers to joint pain NRS. Participants were asked: `over the past four weeks, how intense was your average pain or the average aching in your most bothersome joint,’ where 0 is ‘no pain’, and 10 is ‘pain as bad as could be’? Pain NRS was also categorised as either in the range 0 to 3, or ≥ 4, corresponding to acceptable or unacceptable pain, based on the Patient Acceptable Symptom State (PASS) threshold [15]. PASS represents the threshold of pain which a patient would accept for the remainder of their life.

#### Co-variables

Age (in years), sex (male/female), and BMI class (underweight/normal/pre-obese/obese). Weight (kg), and height (m) were obtained from questionnaire self-report, and BMI (kg/m^2^) was calculated. BMI was then classified using WHO categories [16]; obese sub-categories were collapsed into a single ‘obese’ category of BMI > 30. BMI was treated as categorical because those regarded as either underweight or obese might be less healthy than those with normal BMI. Cases with any missing data were excluded in regression analysis(Pain NRS *n* = 73, BMI *n* = 12 at baseline resulting *n* = 84 cases).Of those with missing pain data 71 (97%) were non-frail at baseline.

### Statistical analysis

Data were summarised using means and standard deviation for normally distributed continuous variables, medians and IQRs for non-normally distributed variables, and n (%) for dichotomous variables. Normality was assessed graphically using histograms and statistically using the Shapiro–Wilk test. Differences between groups were evaluated using Student *t*-tests or Mann–Whitney U tests for continuous variables, and Chi-squared test for categorical variables and Fisher’s exact test when < 5 in a category.

Multivariable cross-sectional analyses investigated associations between joint pain and frailty with the co-variables. Age, sex, and BMI were selected *a priori*, other co-variables were included if *p* < 0.05 in prior bivariate analysis. Categorical variables were classified as a binary outcome (absent/present) in all logistic regression models. Continuous variables were used in all linear regression models. All multivariable statistical models were adjusted for the same co-variables. Transitions in frailty were calculated by comparing baseline frailty classification with 1-year, and mean pain was calculated for those who did transition and compared with those who did not. Transitions between PASS pain categories (NRS < 4 or NRS ≥ 4) between baseline and 1-year were determined within frail and non-frail categories.

Two-wave cross-lagged path modelling permitted simultaneous exploration of plausible causal pathways in non-experimental data compared to independent exploration of association; joint pain and frailty were adjusted for sex, age, and BMI at both baseline and 1-year. The use of standardised regression coefficients permitted comparisons of the strengths of the paths. As frailty was categorical, the model used maximum likelihood estimation, which did not produce root mean square error of approximation (RMSEA) values [17]. Effect size for cross-lagged path modelling, using standardised regression coefficients were interpreted as follows: 0.03 indicates a small effect, 0.07 a moderate effect and 0.12 a strong effect [18].

Statistical analysis was undertaken using STATA SE v16 (StataCorp LLC) and Mplus v8.5 (Muthén & Muthén). A significance level of 0.05 was determined for all statistical tests.

## Results

Data from 1,179 participants who met eligibility criteria were examined. The median age was 73 (range 60–95) years, and 628 (53%) were female. At baseline, 176 (15%) were classified as frail, 1060 (90%) reported pain NRS ≥ 1, and 816 (74%) reported pain NRS ≥ 4 (Table 1). Mean (SD) NRS at baseline was 5.2 (2.5). Distributions of individual FRAIL components and summary of the reported criteria are given in Additional Tables 1 and 2, respectively. The unadjusted association between each FRAIL item and pain NRS is shown in Additional Table 3.

**Table 1:** Characteristics of IMH&W participants at baseline and their bivariate association with frailty classification and pain

Variable	All participants *N* = 1,179	Non-frail *N* = 1,003	Frail *N* = 176	Bivariate association with frailty, OR (CI)	Bivariate association with joint pain NRS, β (CI)
Sex					
Female, n (%)	628 (53)	502 (50)	126 (72)	2.51 (1.77, 3.57), *p* < 0.001	0.86 (0.57, 1.15), *p* < 0.001
Male, n (%)	551 (47)	501 (50)	50 (28)	Ref	Ref
Age (years), median (IQR)	73 (69–78)	73 (69–78)	73 (69–79)	1.005 (0.98, 1.03), *p* = 0.716	-0.02 (-.04, -0.002), *p* = 0.034
Ethnicity					
White, n (%)	1,165 (99)	995 (99)	170 (97)	Ref	Ref
Non-white	13 (1)	8 (1)	5 (3)	3.65 (1.18, 11.31) *p* = 0.024	1.24 (-.16, 2.64), *p* = 0.083
BMI Class^a^, n (%)					
Underweight	17 (1)	13 (1)	4 (2)	3.25 (1.00, 10.58), *p* = 0.049	-0.005 (-1.22, 1.21), p = 0.994
Normal	371 (32)	339 (34)	32 (19)	Ref	Ref
Pre-obese	452 (39)	399 (40)	53 (31)	1.41 (0.89, 2.23), *p* = 0.147	0.32 (-0.28, 0.66), *p* = 0.071
Obese	327 (28)	244 (25)	83 (48)	3.60 (2.32, 5.59), *p* = < 0.001	1.29 (0.92, 1.66), *p* < 0.001
Joint Pain (NRS)					
mean (SD)	5.2 (2.5)	4.8 (2.4)	7.4 (1.7)	1.79 (1.62, 1.98), *p* < 0.001	NA
median (IQR)	5 (3–7)	5 (3–7)	8 (6–8)		
Pain category, n (%)					
Acceptable (NRS 0–3)	290 (26)	288 (31)	2 (1)	Ref	NA
Unacceptable (NRS ≥ 4)	816 (74)	646 (69)	172 (99)	38.46 (9.48, 156.09), *p* < 0.001	NA
Indices of multiple deprivation decile, median (IQR)	8 (5–9)	8 (5–9)	8 (5–9)	*p* = 0.2392 at all levels	*p* = 0.2114 at all levels
Route of recruitment, n (%)					
General Practitioner	1072 (91)	913 (91)	159 (90)	Ref	Ref
Previous studies	101 (8.5)	86 (8.6)	15 (8.4)	1.00, (0.56, 1.78), *p* < 0.996	0.06 (-0.45, .58), *p* = 0.805
Other	6 (0.5)	4 (0.4)	2 (1.1)	2.87 (0.52, 15.81), *p* = 0.226	1.32 (-0.66, 3.30), *p* = 0.191

*Abbreviations*: *SD* standard deviation, *IQR* interquartile range, *NRS* numerical rating scale 0–10, *BMI* body mass index, *OR* odds ratio, *CI* 95% confidence intervals, *β* Beta coefficient, *Ref* reference, *NA* not applicable. Number of observations for each variable vary

^a^WHO classification for BMI (kg/m2), underweight < 18.5, normal 18.5–24.9, pre-obese 25–29.9 and > 30 obese. Missing data at baseline by category: Frailty = 0; Sex = 0 Age = 0; NRS = 75; BMI = 12; Ethnicity = 1

### Cross-sectional associations at baseline

**Fig. 1:**
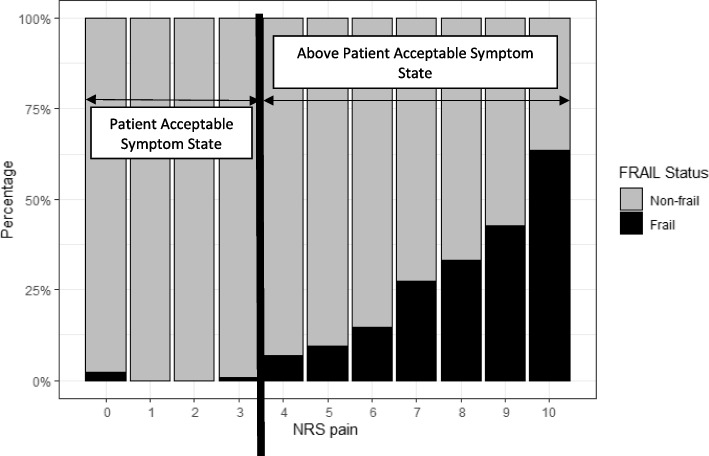
Distribution of baseline joint pain scores by FRAIL classification including Patient Acceptable Symptom State threshold (*n* = 1106).Abbreviation: NRS – pain numerical rating scale (0–10)

In bivariate analyses, greater pain intensity, female sex, and obesity were associated with frailty (Fig. 1, Table 1). Female sex, and obesity were associated with joint pain NRS (Table 1). Pain NRS ≥ 4 was reported by almost all participants classified as frail at baseline (Fig. 1). Females reported more severe pain (mean (SD) pain NRS = 5.59 (2.47)) than did males (4.73 (2.37), *p* < 0.001), and obesity was associated with higher joint pain NRS compared to normal BMI (r_s_ = 0.20, *p* < 0.001)). There was an association between higher BMI class and frailty, with a higher proportion of frail participants who were obese 82 (48%) than in the lower BMI classes (*X*^2^ = 44.05, *p* < 0.001). In multivariable regression, higher pain NRS, female sex, BMI class and age were associated with frailty at baseline (Table 2).

**Table 2:** Associations of joint pain and other characteristics at baseline with frailty at baseline and 1-year (*N* = 1095)

		Frailty	
		Baseline	1-year
Baseline Factor	Interval/category	aOR (95%CI)	aOR (95%CI)
Frailty	(non-frail, frail)	NA	13.24 (8.43, 20.80), *p* < 0.001
Joint Pain	Pain (NRS 0/10)	1.72 (1.56, 1.92), *p* < 0.001	1.28 (1.15, 1.43), *p* < 0.001
Sex	Male	Ref	Ref
	Female	1.81 (1.22, 2.68), *p* = 0.003	1.39 (0.89, 2.17), *p* = 0.15
Age	Years	1.03 (1.00, 1.06), *p* = 0.026	1.04 (1.01, 1.08), *p* = 0.006
BMI Class^a^	Underweight	3.30 (0.83, 13.08), *p* = 0.089	0.25 (0.02, 2.47), *p* = 0.233
	Normal	Ref	Ref
	Pre-obese	1.48 (0.89, 2.46), *p* = 0.129	1.41 (0.79, 2.47), *p* = 0.251
	Obese	2.69 (1.63, 4.42), *p* < 0.001	2.96 (1.66, 5.27), *p* < 0.001
Pseudo r^2^		0.2259	0.3409

The outcome measure is frailty (binary). The baseline model is frailty with pain adjusted for age, sex, and BMI class. The 1-year model is 1-year frailty adjusted for baseline factors of frailty, pain, age, sex, and BMI class

*Abbreviations*: *NRS* numerical rating scale (0–10), *BMI* Body Mass Index, *aOR* adjusted odds ratio**,** *CI* 95% confidence intervals, *Ref* reference group, *NA* Not Applicable

^a^WHO classification for BMI (kg/m2), underweight < 18.5, normal 18.5–24.9, pre-obese 25–29.9 and > 30 obese

### Joint pain and frailty changes and transitions between baseline and 1 year

The proportion of participants classified as frail, mean pain severity and proportions reporting pain NRS ≥ 4 changed little between baseline and 1-year. At 1-year, 163 (14%) participants were classified as frail, 1060 (96%) reported pain NRS ≥ 1, and 816 (74%) pain NRS ≥ 4 whilst mean (SD) pain NRS was 5.0 (2.5). However, 127 (11%) participants had changed their frailty status between baseline and 1-year; 58 (5%) transitioned from non-frail to frail and 69 (6%) transitioned from frail to non-frail. 215 (21%) participants transitioned between pain acceptability categories. 93 (9%) people transitioned from pain NRS < 4 at baseline to pain NRS ≥ 4 at 1-year, and 122 (12%) transitioned from pain NRS ≥ 4 to pain NRS < 4 (Additional Table 4).

People who transitioned from non-frail to frail classification reported more severe pain at baseline (NRS mean 6.4 (95%CI 5.8, 7.1) than those who remained non-frail (NRS mean 4.7 (95%CI 4.5, 4.8). Each unit increase in pain NRS at baseline was associated with a greater risk of becoming frail at 1-year [OR 1.42 (95%CI 1.25, 1.63), *p* =  < 0.001]. However, no significant difference in baseline pain severity was found between those who transitioned from frail to non-frail classification and those who remained frail (NRS mean 7.2 (95%CI 6.8, 7.5) and 7.5 (95%CI 7.1, 7.9), *p* = 0.187) (Additional Table 4). In people who were frail at baseline, lower pain did not significantly predict the likelihood of becoming non-frail [OR 1.13 (95%CI 0.94, 1.35) per unit increase in pain NRS at baseline, *p* = 0.188].

People who transitioned from unacceptable (NRS ≥ 4) to acceptable (NRS < 4) pain were less likely to be frail at baseline (*n* = 11, 9%) than were those who continued with unacceptable pain (*n* = 157, 24%, *X*^2^ = 13.57, *p* < 0.001). Of the (*n* = 92, 9%) people who transitioned from acceptable (NRS < 4) to unacceptable (NRS ≥ 4) pain one person was frail at baseline and five at 1-year. Due to low numbers this was not analysed further. Unacceptable (NRS ≥ 4) pain at 1-year was predicted by baseline unacceptable (NRS ≥ 4) pain (aOR 6.54 (95%CI 4.67, 9.15), *p* < 0.001) and baseline frailty classification (aOR 2.76 (95%CI 1.46, 5.21), *p* = 0.002), each adjusted for age, sex, and BMI.

### Longitudinal associations of baseline variables with joint pain and frailty at 1-year

Unadjusted analysis showed each unit of baseline pain was associated with increased risk of 1-year frailty classification [OR 1.61 (95%CI 1.47, 1.77), *p* < 0.001]. In multivariable regression, baseline pain remained associated with 1-year frailty classification (aOR 1.28 (95%CI 1.15, 1.43), *p* < 0.001 adjusted for baseline frailty, sex, age, and BMI (Table 2).

Unadjusted bivariate analysis showed frailty at baseline was associated with more severe pain at 1-year β = 2.01 (95%CI 1.62, 2.39), *p* < 0.001. In multivariable regression, baseline frailty remained associated with 1-year pain severity β = 0.56 (95%CI 0.50 to 0.61), *p* =  < 0.001 adjusted for baseline pain, sex, age, and BMI (Table 3).

**Table 3:** Associations of frailty at baseline and other characteristics with joint pain at baseline (*N* = 1095) and 1-year (*N* = 1004)

		Pain	
		Baseline (*n* = 1095)	1-year (*n* = 1004)
Baseline Factor	Interval/category	β Coef (95%CI)	β Coef (95%CI)
Frailty	(non-frail, frail)	2.29 (1.91, 2.66), *p* < 0.001	0.39 (0.04, 0.75), *p* = 0.027
Pain	Pain (NRS) (0–10)	NA	0.56 (0.50, 0.61), *p* < 0.001
Sex	Male	Ref	Ref
	Female	0.63 (0.36, 0.90), *p *< 0.001	0.33 (0.09, 0.58), *p* = 0.008
Age	Years	-0.01 (-0.03, 0.01), *p* = 0.226	0.00 (-0.02, 0.02), *p* = 0.783
BMI Class^a^	Underweight	-0.48 (-1.60, 0.65), *p* = 0.408	-0.55 (-1.63, 0.52), *p* = 0.312
	Normal	Ref	Ref
	Pre-obese	0.33 (0.00, 0.65), *p* = 0.047	0.27 (-0.02, 0.56), *p* = 0.069
	Obese	0.93 (0.58, 1.29), *p* < 0.001	0.55 (0.23, 0.87), *p* = 0.001
r^2^		0.1829	0.3747

The outcome measure is pain (continuous variable). The baseline model is pain with frailty adjusted for age, sex, and BMI class. The 1-year model is 1-year pain with baseline frailty adjusted for baseline factors of pain, age, sex, and BMI class

*Abbreviations*: *NRS* numerical rating scale (0–10), *BMI* Body Mass Index, *OR* odds ratio, *95%CI* confidence intervals, *NA* not applicable, *Ref* reference group

^a^WHO classification for BMI, underweight < 18.5, normal 18.5–24.9, pre-obese 25–29.9 and > 30 obese

**Fig. 2:**
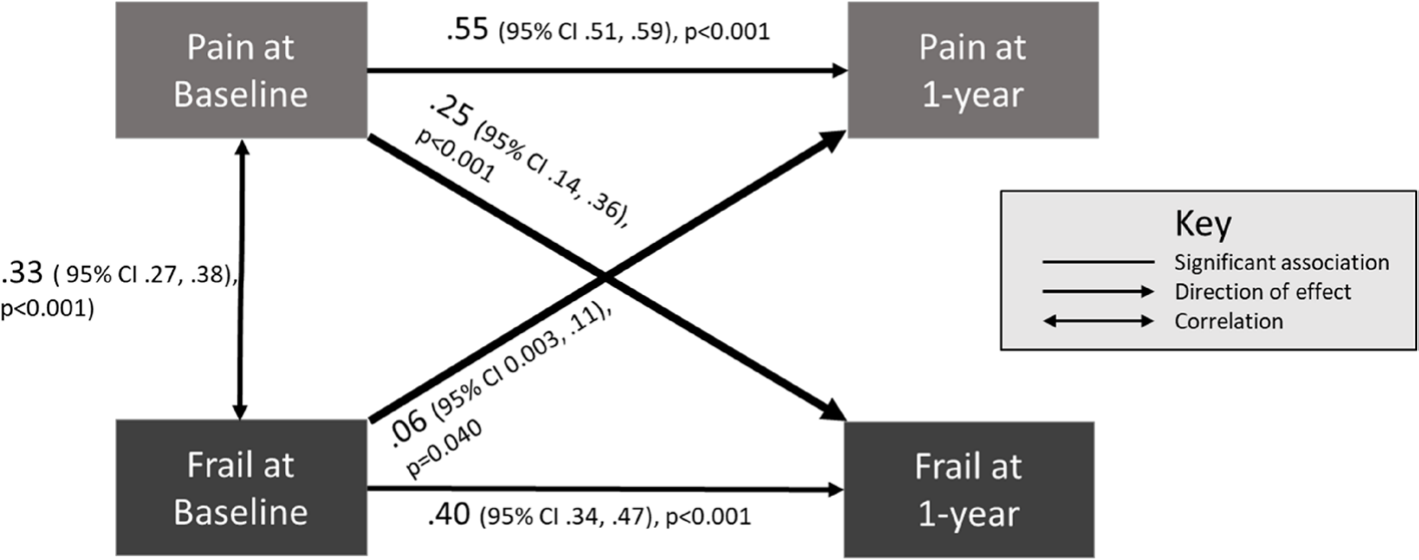
Cross-lagged path analysis model showing standardised regression coefficients of the relationship between joint pain and frailty at baseline and 1-year, adjusted for age, sex and BMI (*n* = 995) Abbreviation: CI – 95% confidence intervals

To determine whether these findings in bivariate and multivariable models might indicate bidirectional relationships between pain and frailty we undertook cross-lagged path analysis, including both pain and frailty both at baseline and 1-year, together with covariates of age, sex, and BMI (Fig. 2, Additional Table 5). Pain at baseline predicted higher 1-year pain [β = 0.55, (95%CI 0.51, 0.59), *p* =  < 0.001], and frailty classification at baseline predicted 1-year frailty [β = 0.40, (95%CI 0.34, 0.47) *p* =  < 0.001] (Fig. 2). There was a strong effect of higher pain at baseline predicting 1-year frailty [β = 0.25, (95%CI 0.14, 0.36) *p* =  < 0.001], and a small to moderate effect frailty at baseline predicting higher 1-year pain [β = 0.06, (95%CI 0.003, 0.11), *p* = 0.040].

## Discussion

We found that joint pain was strongly associated with current and future frailty in the IMH&W cohort, even after adjusting for baseline age, sex, BMI, and frailty status. Greater pain severity increased the risk of transitioning from non-frail to a frail state over one-year of follow-up but did not appear to be a significant barrier to the transition from frail to a non-frail state over the same timeframe. Additionally, we observed a small to moderate association between frailty classification and future joint pain, over one-year. These findings support the hypothesis that the relationship between joint pain and frailty is bidirectional.

Our findings have confirmed the directional pathway that baseline pain is predictive of future frailty [19–22], and people can transition between frailty and non-frail classifications [3]. In contrast to previous studies [19], we were able to demonstrate frailty predicts future pain, with a small to moderate effect. Such a bidirectional relationship implies pain and frailty could act together in a vicious cycle in which each accelerates the development of the other. Our findings are of considerable clinical importance given the strength of these predictive relationships and the fact almost all (99%) participants in our study classified as frail rated their pain at a level regarded as unacceptable.

Our findings are of importance given expert opinion [23] and current advice provided by NHS England [24] and NICE guidelines [25] about frailty prevention do not mention the role of pain. Exercise and nutrition have to date been the primary interventions employed in studies aiming to prevent or reverse frailty [26–28]. The absence of interventions to address pain could partly explain why the prevention and management of frailty remains a challenge. Our findings justify the inclusion of pain reducing strategies within interventions designed to prevent, delay, or manage frailty. Furthermore, pain is not widely recognised as a feature or complication of frailty [29] nor widely used as an outcome measure in frailty studies [26–28]. Our findings justify the inclusion of pain as an outcome of importance in frailty studies.

Whilst musculoskeletal conditions are linked with pain, fatigue, physical activity, obesity, and comorbidities [7], many of which are included in frailty measures, frailty is rarely mentioned as a complication of musculoskeletal conditions. The NICE guidelines for the treatment and management of chronic pain make no mention of frailty [30]. The only reference to frailty from the Core Standards for Pain Management Services in the UK [31] is in terms of specialist palliative care. Our findings suggest raised awareness of the risks associated with pain and frailty could benefit public health interventions, and the management of these conditions by medical professionals and social care. Identifying people at risk of frailty, for example because they have chronic pain, alongside those who may benefit from an intervention is key to addressing future health challenges.

Our study had several strengths and some weaknesses. We included in our analysis’s co-variables such as age, sex and BMI previously linked to both pain and frailty, and which could otherwise have introduced confounding. Previous research has frequently focussed on single-sex cohorts [14, 19, 32]. Although, FRAIL has been used worldwide, but in the UK has been reported only for older British men [33]. Our cohort was not an epidemiologically representative sample of the population, and the relatively high prevalence of joint pain and frailty in our sample reflect our recruitment processes. Whilst this means our study cannot derive the population prevalence of pain or frailty, this sampling issue would not affect the validity of or analysis of the relationships between pain and frailty. In the ascertainment of frailty, our methods used self-report data which may be subject to recall bias [34, 35]. Whilst this may have introduced some classification errors, we believe such errors would most likely reduce the precision of our results rather than introduce systematic bias. Our methods for identifying frailty were limited, as are all measures of frailty, as there is currently no direct measure of vulnerability to challenge. Other frailty classification tools utilise clinical measures, however, FRAIL has been shown to perform comparably with other tools [36, 37]. This study explored the relationship with joint pain, and it may be this aspect of pain has a stronger relationship with frailty than those previously tested due to its relationship with weight-bearing and mobility. Previous studies exploring the relationship between pain and frailty have been limited by using categorical indicators of pain (yes/no), whereas we used a validated, continuous measure which enabled us to identify a dose–response (e.g., greater pain, greater risk of frailty). As a unidimensional measure of pain NRS has limitations, however, pain management focusses on the overall reduction in pain rather than dimensional aspects. Other covariables, for example, cognitive impairment may be related to frailty and pain but were not measured in this study. Polypharmacy may also be associated with frailty and pain but will be too closely correlated to the comorbidities count to be an independent variable.

Our crossed-lagged path analysis used two timepoints, and our findings could be in the future be strengthened with additional timepoints, which could potentially add to the findings. The advantage of cross-lagged methods is that they take account of baseline and 1-year factors within the same model. While path analysis is regarded as indicative of directional pathways, it is not conclusively causal in observed non-experimental data. We recognise one-year is a relatively short period of time to observe changes in frailty: stronger relationships between pain and frailty might have been observed had we used a longer period of follow-up. However, we did observe change over one-year which align with the findings of other longitudinal studies [3].

Further research should identify pain mechanisms through which pain predicts frailty to identify people at risk of frailty and develop interventions to reduce the risk of future frailty while addressing current pain. Interventional studies are needed to assess feasibility, acceptability, and efficacy. Our findings suggest that frailty is potentially reversible, at least to an extent, raising hope to enable people to age well.

In conclusion, there is a bidirectional relationship between pain and frailty which could lead to a vicious cycle in which each accelerates the other’s progression. This justifies attempts to prevent frailty by addressing pain, and to include pain measures as outcomes in frailty studies.

## Supplementary Information

**Additional file 1:** The bidirectional relationship between chronic joint pain and frailty: data from the Investigating Musculoskeletal Health and Wellbeing cohort.

## Data Availability

The data that support the findings of this study are available from the authors upon reasonable request and with permission of the University of Nottingham. Enquires should be sent to David.Walsh@nottingham.ac.uk.
