# Supplementary material for: The bidirectional relationship between chronic joint pain and frailty: data from the Investigating Musculoskeletal Health and Wellbeing cohort
Source: BMC Geriatr. 2023 May 5;23:273. doi: 10.1186/s12877-023-03949-4 (PMC10161600; doi:10.1186/s12877-023-03949-4)

The bidirectional relationship between chronic joint pain and frailty: data from the Investigating Musculoskeletal Health and Wellbeing cohort.

Additional Tables

## Additional table 1. Frequency of responses to items meeting FRAIL criteria (n=1,179).

| FRAIL | Total, n (%) | Non-frail, n (%) | Frail, n (%) |
| --- | --- | --- | --- |
| Fatigue | 287 (24) | 130 (13) | 157 (89) |
| Resistance | 366 (31) | 197 (20)136 (14) | 163 (93) |
| Ambulation | 299 (25) | 137 (14) | 171 (97) |
| Illness | 29 (3) | 7 (1) | 22 (13) |
| Loss of weight | 180 (15) | 122 (12) | 58 (33) |

Total non-frail =1003, frail = 176

The percentage is the proportion of a group who reported meeting the FRAIL criterion. For example, 287 (24%) of participants reported feeling tired all or most of the time. Of the 1003 people classified as non-frail 130 (13%) reported feeling tired all or most of the time, and of the 176 people classified as frail 157 (89%) reported feeling tired all or most of the time. Participants are classified as frail if they meet ≥3 criteria.

## Additional table 2. Summary of the number of FRAIL criteria reported (n=1,179).

| FRAIL criteria | Baseline, n (%) | | 1-year, n (%) | |
| --- | --- | --- | --- | --- |
| 0 | | 579 (49) | | 592 (50) |
| 1 | | 258 (22) | | 238 (20) |
| 2 | | 166 (14) | | 184 (16) |
| 3 | | 135 (11) | | 139 (12) |
| 4 | | 39 (3) | | 22 (2) |
| 5 | | 2 (0.2) | | 4 (0.3) |

Participants are classified as frail if they meet ≥3 criteria.

## Additional table 3. The unadjusted association between FRAIL items and pain NRS (n=1106)

| FRAIL Item | Spearman rho | | OR (95% CI) | | | P value |
| --- | --- | --- | --- | --- | --- | --- |
| Fatigue | 0.38 |  | | 1.51 (1.41, 1.62) | <0.001 | |
| Resistance | 0.44 |  | | 1.69 (1.56, 1.83) | <0.001 | |
| Ambulation | 0.46 |  | | 1.66 (1.54, 1.79) | <0.001 | |
| Illnesses | 0.08 |  | | 1.25 (1.06, 1.48) | 0.008 | |
| Loss of weight | 0.08 |  | | 1.10 (1.03, 1.18) | 0.005 | |

Abbreviations: CI – confidence interval; OR – odds ratio;

## Additional table 4. Transitions of frailty status and NRS pain at baseline and one-year.

| Baseline | 1-Year | Total, n (%) | Frail at baseline, n (%) | Non-frail at baseline, n (%) | Frail at 1-year, n (%) | Non-frail at 1-year, n (%) | NRS  Mean (95%CI) | p value | |
| --- | --- | --- | --- | --- | --- | --- | --- | --- | --- |
| Frailty status | |  |  |  |  |  |  |  |  |
| Non-frail | Frail | 57 (5) | - | - | - | - | 6.44 (5.8, 7.1) | <0.001 |  |
| Non-frail | Non-frail | 945 (80) | - | - | - | - | 4.68 (4.5, 4.8) |  |  |
| Frail | Non-frail | 69 (6) | - | - | - | - | 7.15 (6.8, 7.5) | 0.1872 |  |
| Frail | Frail | 107 (9) | - | - | - | - | 7.51 (7.2, 7.9) |  |  |
| Pain category | |  |  |  |  |  |  |  |  |
| NRS<4 | NRS<4 | 145 (14) | 1 (1) | 144(99) | 5 (3) | 140 (97) |  | NA |  |
| NRS<4 | NRS≥4 | 93 (9) | 1 (1) | 92 (99) | 5 (5) | 88 (95) |  |  |  |
| NRS≥4 | NRS<4 | 122(12) | 11 (9) | 111 (91) | 6 (5) | 116 (95) |  | <0.001 |  |
| NRS≥4 | NRS≥4 | 655 (65) | 159 (24) | 496 (76) | 145 (22) | 510 (78) |  |  |  |
|  |  |  |  |  |  |  |  |  |  |

Abbreviations: NRS – numerical rating scale (0-10); CI – Confidence intervals.

Note there are two t-tests: firstly, for people non-frail at baseline who transitioned to frailty compared to those who remained non-frail. Secondly, for people frail at baseline who transitioned to non-frailty compared to those who remained frail.

There were too few people with NRS<4 and frail to examine a statistical association. People with NRS≥4 at baseline and frailty at baseline *X*^2^= 14.00

## Additional table 5. Standardised beta coefficients of pathway analysis.

| To | From | Std β Coef | **95% C**onfidence Intervals | | **p**-value |
| --- | --- | --- | --- | --- | --- |
| NRS1 | FRAIL0 | 0.06 | 0.01 | 0.10 | 0.040 |
| NRS1 | NRS0 | 0.55 | 0.51 | 0.59 | <0.001 |
| NRS1 | SEX | 0.07 | 0.03 | 0.11 | 0.009 |
| NRS1 | AGE | 0.01 | - 0.03 | 0.05 | 0.069 |
| NRS1 | BMI1 | 0.09 | 0.05 | 0.14 | <0.001 |
|  |  |  |  |  |  |
| FRAIL1 | FRAIL0 | 0.40 | 0.35 | 0.46 | <0.001 |
| FRAIL1 | NRS0 | 0.25 | 0.16 | 0.34 | <0.001 |
| FRAIL1 | SEX | 0.06 | - 0.02 | 0.14 | 0.194 |
| FRAIL1 | AGE | 0.12 | 0.05 | 0.20 | 0.008 |
| FRAIL1 | BMI1 | 0.15 | 0.07 | 0.22 | 0.001 |
|  |  |  |  |  |  |
| BMI1 | BMI0 | 0.93 | 0.92 | 0.94 | <0.001 |
| BMI1 | SEX | - 0.00 | - 0.02 | 0.02 | 0.871 |
| BMI1 | AGE | - 0.04 | - 0.05 | - 0.02 | 0.002 |
|  |  |  |  |  |  |
| BMI0 | SEX | - 0.04 | - 0.09 | 0.01 | 0.235 |
| BMI0 | AGE | - 0.16 | - 0.21 | - 0.11 | <0.001 |
|  |  |  |  |  |  |
| SEX | AGE | - 0.04 | - 0.09 | 0.01 | 0.225 |
|  |  |  |  |  |  |
| NRS0 | SEX | 0.20 | 0.15 | 0.25 | <0.001 |
| NRS0 | AGE | - 0.03 | - 0.08 | 0.02 | 0.338 |
| NRS0 | BMI0 | 0.19 | 0.14 | 0.24 | <0.001 |
|  |  |  |  |  |  |
| FRAIL0 | SEX | 0.16 | 0.11 | 0.21 | <0.001 |
| FRAIL0 | AGE | 0.06 | 0.01 | 0.11 | 0.050 |
| FRAIL0 | BMI0 | 0.22 | 0.17 | 0.27 | <0.001 |
|  |  |  |  |  |  |
| NRS0* | FRAIL0* | 0.33 | 0.28 | 0.37 | <0.001 |

*Note: pathway NRS0 and FRAIL0 is a correlation and therefore bidirectional.

Abbreviations: NRS- numerical rating scale; BMI – body mass index; the suffix number refers to the timepoint 0 = baseline and 1= 1-year.

Additional Figures

## Additional Figure 1. IMH&W CONSORT Flow Diagram


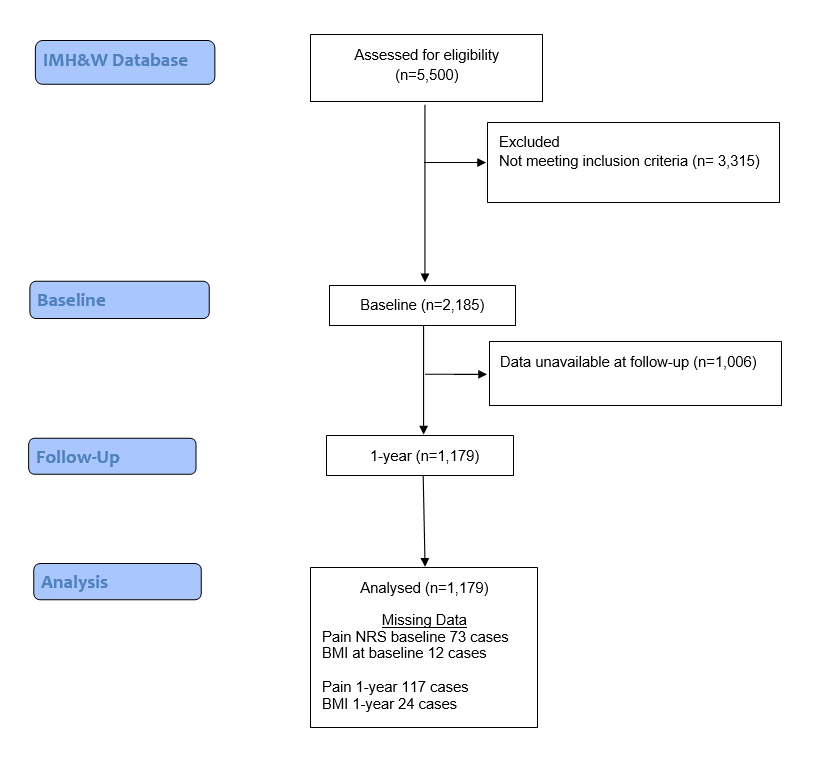

Supplement: Supplementary file 1 — Additional file 1: The bidirectional relationship between chronic joint pain and frailty: data from the Investigating Musculoskeletal Health and Wellbeing cohort. [file 12877_2023_3949_MOESM1_ESM.docx]
